# Design of CoNiCrFeCu-xSc High-Entropy Alloy Fillers for Braze-Welding of WC-Co to Steel

**DOI:** 10.3390/ma19081606

**Published:** 2026-04-16

**Authors:** Peiquan Xu, Shicheng Sun, Benben Li, Leijun Li

**Affiliations:** 1 Shanghai Collaborative Innovation Center of Laser Advanced Manufacturing Technology, School of Materials Science and Engineering, Shanghai University of Engineering Science, Shanghai 201620, China; ssc2747434849@163.com (S.S.); lbb15956740472@163.com (B.L.); 2 State Key Laboratory of Materials for Advanced Nuclear Energy, Shanghai University, Shanghai 200444, China; 3 Department of Chemical and Materials Engineering, University of Alberta, Edmonton, AB T6G 2V4, Canada

**Keywords:** high-entropy alloys (HEAs), scandium, hard metal, wetting behavior, HRTEM

## Abstract

Efficient joining of hard metals to steels is crucial for supporting sustainable manufacturing under emissions strategies to minimize CO_2_. CoNiCrFeCu high-entropy alloy containing scandium (Sc) was designed as a filler for laser braze-welding of WC-Co and steel. The designed compositions with different Sc levels were melted and cast in a high-vacuum non-consumable arc furnace. The results showed that the as-cast microstructure was a complex mixture of a networked Ni_2_Si, elongated Cr-Fe-Co solid-solution phase, and Fe-Ni-Co-Cu solid-solution phase. Scandium was shown to have formed compounds with nickel/cobalt and copper. The TG-DSC analysis confirmed that the melting points of the designed compositions were between 973.7 °C and 981.5 °C. The maximum spreading area of the CoNiCrFeCu-0.9Sc composition on AISI 1045 steel was 64.83 mm^2^, and on the WC-Co cermet it was 78.63 mm^2^. The interface between the fusion zone and AISI 1045 steel exhibited an epitaxial growth of dendrites from the steel base metal. The interface between WC-Co and the fusion zone exhibited a partial penetration of brazing filler into the Co matrix, forming a metallurgical bonding between the dissimilar materials. Sc, as an alloying element in the filler metal, enhanced the bond formation because it decreased the solidus temperature and increased wetting.

## 1. Introduction

In the early 2000s, an approach for designing multi-principal element alloys with equimolar or near-equimolar ratios was proposed [1]. These alloys have been known as high-entropy alloys (HEAs), characterized by incorporating five or more elements in equimolar or near-equimolar ratios, with each element’s concentration ranging from 5% to 35%. HEAs exhibit the high-entropy effects [2], sluggish effects [3], lattice distortion effects, and cocktail effects [4,5]. George et al. [6] confirmed that the high mixing entropy in HEAs enabled the formation of a single, stable solid solution, which allowed for better structural stability and superior mechanical properties, such as toughness.

HEAs were used as brazing filler metals for joining dissimilar materials [7], particularly between hard materials (e.g., carbides and ceramics) and ductile metals (e.g., steels) [8]. Cemented carbides [9,10] and their joining to steels [11,12] has been achieved using several processes, including: brazing [13], diffusion bonding [14], arc brazing [15], friction stir welding [16], laser welding [17], electron beam welding [18,19], resistance welding [20], combustion welding [21], laser powder bed fusion [22], and capacitor discharge welding [23]. Among all joining methods, brazing has been the most promising and popular. A significant challenge in brazing WC-Co to steels is the poor joint strength [24]. Exploring and developing new brazing filler metals has been active [25]; for example, Tillmann et al. [26] investigated the brazing of WC-Co and steel using a non-flux procedure with special filler metals that showed remarkable wetting properties. HEAs were designed as fillers for dissimilar joining of Ti_3_SiC_2_/Cu [27], Al_2_O_3_/Ti6Al4V [28], GH3536/SS304 [29], ZrB_2_-SiC/Nb [30], TiAl/TC4 [31], tungsten/steel [32], TiAl [33], SiC [34], and CoSb_2.75_Sn_0.05_Te_0.20_/Cu [35].

Ding et al. [36] successfully used high-entropy CoCrFeMnNi alloy brazing of filler metals to diffusion-braze Cu/304 stainless steel, and no brittle intermetallic compounds were found at the interface. Bridges et al. [37] effectively employed NiMnFeCoCu high-entropy alloy brazing of filler metals for the joining of nickel-based superalloys, resulting in a fourfold increase in the hardness of the brazed HEAs. Chen et al. [38] innovatively utilized Ti_5_Fe_5_Al_30_Ni_30_Cu_30_ and Ti_10_Fe_10_Cr_5_Ni_35_Cu_40_ HEAs for resistance welding of TA2/Q235 and conducted in-depth correlation analysis between joining process parameters and joint microstructure. Tillmann et al. [39] employed eutectic HEAs brazing filler Nb_0.73_CoCrFeNi_2.1_ to successfully braze Crofer 22 APU alloy and Hf-metallized 3YSZ ceramic. In these joints, the eutectic microstructure consisted of a Laves phase and a solid-solution phase, imparting high strength and extraordinary ductility to the joints.

Recently, refractory high-entropy alloys (RHEAs), mainly based on refractory elements (e.g., Cr, W, and Re), became attractive in high-temperature applications due to their high strength, good oxidation resistance, and outstanding corrosion resistance [40]. The addition of rare earth was believed to enhance the deformability and the strength through mechanisms such as solid-solution strengthening and precipitation strengthening [41].

Research by Mousavi et al. [42] showed that adding the rare earth Sc could effectively refine grain structures and strengthen grain boundaries. A significant reduction in porosity and an increase in density following the introduction of rare earth elements were found by Deng et al. [43].

Application of innovatively designed high-entropy alloys as a brazing filler can utilize their inherent high-entropy effect and sluggish effect, which may give the fillers distinct thermodynamic and kinetic properties. The objective of the present research is to develop a high-entropy alloy, CoNiCrFeCu-xSc, and explore the possibilities of its application as a brazing filler metal in the joining of hard alloys to steel. We focus on studying the influence of the rare earth element scandium (Sc) on the melting point and wetting behavior of the CoNiCrFeCu-xSc HEAs. The microstructure, phase composition, and crystal structure of the CoNiCrFeCu-xSc HEAs will be disclosed. The relationship between the brazing filler metal and the weld formation, and the bend strength of laser-brazed joints, will be addressed.

## 2. Materials and Methods

### 2.1. The Preparation of CoNiCrFeCu-xSc HEAs

The as-cast CoNiCrFeCu-xSc HEAs were produced using a high-vacuum non-consumable arc melting furnace. The chemical compositions (wt.%) of CoNiCrFeCu-xSc high-entropy alloys are shown in Table 1. The arc melting process includes the following:

(1) Weighing the raw material particles to be melted according to Table 1. The raw materials were all in block or rod form and had a purity greater than 99.99%. The impurities were less than 0.01% of sulfur and phosphorus. Cobalt blocks were 3–15 mm in length, chromium was 3–10 mm in length, iron was 3 × 3 mm, nickel was 3 × 3 mm, copper was 3 × 3 mm, and scandium was 3–10 mm in length.
(2) Vacuuming the high-vacuum non-consumable arc melting furnace (DHL500, Beijing, China), and back-filling with an argon gas of 99.99% purity until the gauge pressure in the furnace was −0.05 MPa. The vacuum level during melting was 10^−4^ Pa.
(3) Melting the raw materials (ingot) repeatedly for 4 to 6 times. The duration of each melting was 3 min. An electromagnetic stirrer was used for stirring. The current was 400–600 A. The melt was cooled down to room temperature in a furnace to obtain disk-shaped ingots.

**Table 1:** Chemical composition of CoNiCrFeCu-xSc high-entropy alloys (wt.%).

Elements	Cu	Ni	Cr	Fe	Mn	Si	B	Y	Sc	Co
Co1	16	20	16	16	1	5	3	0.3	0.5	Bal.
Co2	16	20	16	16	1	5	3	0.3	0.7	Bal.
Co3	16	20	16	16	1	5	3	0.3	0.9	Bal.

Small amounts of Si, B, and Y were intentionally added to the CoNiCrFeCu-xSc high-entropy brazing alloy during vacuum melting. Silicon was added to significantly lower the liquidus temperature, enhance melt fluidity, and improve the wettability of the filler alloy. Boron strongly lowers the melting point, markedly enhances wettability, and segregates at grain boundaries to reduce defects and strengthen grain boundaries. Yttrium helps remove oxygen and sulfur, refines the microstructure, improves the interface and high-temperature stability, and works jointly with scandium. These elements are added in small quantities and do not form excessive brittle intermetallic phases. Therefore, they do not negatively affect the overall mechanical properties of the CoNiCrFeCu-xSc. All elements are added in the form of high-purity particles. When charging the materials, the low-melting-point constituents are added first, followed by the high-melting-point constituents; this practice effectively reduces evaporation losses of the low-melting-point materials.

However, excessive Si, B, and Y are harmful. High Si causes brittle Si-rich phases or silicides in the braze seam or interface, which can form continuous brittle layers under poor cooling or solution treatment and reduce joint ductility and toughness. B forms hard, brittle borides with Fe, Cr, and Ni, degrading fatigue and impact performance. Y can combine with O, B, or Si to form complex oxides/borides/silicides; with high B and Si, it may be tied up as large inclusions instead of fine dispersoids.

The disk-shaped ingots were cut into 40 × 4 × 0.5 mm sheets by wire EDM cutting, followed by surface oxidation removal by sandpaper, cleaning, and drying in an oven.

### 2.2. The Melting Points, Wetting Behavior, and Hardness of the CoNiCrFeCu-xSc Filler

The melting point of the CoNiCrFeCu-xSc HEAs with different Sc contents was determined and compared by Thermogravimetry–Differential Scanning Calorimetry (TG-DSC) using a simultaneous thermal analyzer (SDT650, TA Instruments, New Castle, DE, USA). The experimental parameters included the measuring temperature range of 20–1100 °C, heating rate at 20 °C/min, and argon shielding.

To investigate the wetting behavior of the CoNiCrFeCu-xSc HEAs (CoNiCrFeCu-xSc), the samples were cut into cubes (2 × 2 × 2 mm). These samples were polished, and the surface oxide layers were removed before undergoing spreading experiments in a high-frequency induction furnace (HX-GP, Yueqing Hongri Electronic Technology Co., Ltd., Yueqing, China). WC-Co and AISI 1045 steel were used as the substrates for wetting and spreading. The dimensions of both substrates were 20 × 20 × 3 mm.

A digital micro hardness tester (HXD-1000TMC/LCD, Shanghai Taiming Optical Instrument Co., Ltd., Shanghai, China) was used to evaluate the effect of varying scandium (Sc) content on the hardness of the CoNiCrFeCu-xSc HEAs. Experimental parameters included 500 gf load, 15 s dwell time, and random selection of test points for repeat testing.

The wettability of the brazing filler metals on the base material, AISI1045 steel, and WC-Co was evaluated by calculating the spreading area of the CoNiCrFeCu-xSc HEAs on the base materials after the furnace heating with the help of Adobe Photoshop 2023 (Version 24.7) software (Adobe Inc., San Jose, CA, USA). To ensure comparability of the spreading experimental results, each group of furnace heating was repeated four times, and the average values of the results were recorded while keeping all other experimental conditions constant.

### 2.3. Microstructure and Phase Analysis of the CoNiCrFeCu-xSc HEAs

The microstructure and phase constitution of the CoNiCrFeCu-xSc HEAs were characterized by an X-ray diffractometer (X’Pert Pro, Malvern Panalytical B.V., Almelo, The Netherlands), a field emission scanning electron microscope (SEM), and a field transmission electron microscope (TEM).

The XRD measurements were performed on an X’Pert PRO X-ray diffractometer (PANalytical B.V., Almelo, The Netherlands) with Cu Kα radiation (0.15406 nm). Scans were performed from 5° to 80° with a step size of 0.02626°. The scanning speed was 0.4376°/s at a beam current and voltage of 40 mA and 40 kV, respectively.

The metallographic samples were prepared by mounting, grinding, and polishing, and etched with Murakami’s reagent (10 g of potassium ferricyanide K3Fe (CN)6, 10 g of sodium hydroxide NaOH, and 100 mL of water, freshly prepared). The microstructures were examined using a JEM7600F scanning electron microscope (JEOM, Tokyo, Japan).

FTEM samples were prepared using a Gatan 695 ion beam milling instrument (Gatan, Pleasanton, CA, USA). Mechanical grinding to a thickness of 30 μm, followed by ion milling at 4 kV with a beam tilt of ±6° for hole drilling. After hole drilling, milling is performed at 3 kV with a beam tilt of ±3° for 20 min. TEM and high-resolution microstructure of the CoNiCrFeCu-xSc high-entropy alloys were characterized using a FEI Tecnai G2 F20 X-TWIN transmission electron microscope (FEI, Hillsboro, OR, USA), which had STEM imaging with both EDS and EELS analytical analysis capabilities, and a CCD camera for standard TEM imaging.

### 2.4. Application of CoNiCrFeCu-xSc as Filler Metal for Laser Welding–Brazing of WC-Co and AISI 1045 Steel

The CoNiCrFeCu-xSc HEAs were cut into thin sheets with dimensions of 40 × 4 × 0.5 mm using a wire-cutting method. They were then used as brazing filler to produce a WC-20Co/AISI 1045 brazed joint using laser braze-welding. The WC-Co, used as one of the base materials, had a chemical composition of 4.9 C, 20 Co, and 75.1 W (wt.%). AISI 1045 steel was used as the other base material. It had the following chemical composition: 0.45 C, 0.26 Si, 0.61 Mn, 0.0003 S, 0.003 P, 0.25 Cr, 0.26 Cu, and balance Fe (wt.%). Both substrates had the dimensions of 40 × 40 × 3 mm. The brazing filler metal was a 0.5 mm-thick CoNiCrFeCu-xSc HEA designed in this study.

The laser parameters for braze-welding are shown in Table 2. Specimens V_1_, V_2_, and V_3_ were used to explore the parameter window for joint penetration. V_4_, V_5_, and V_6_ were used to investigate the effect of laser scanning speed on the formation of brazed joints. Based on the optimized brazing process parameters, V_6_, V_7_, and V_8_ were used to compare the brazing filler scandium content Co1, Co2, and Co3 on brazing performance, including the wettability, XRD, DSC, micro hardness, and strength of the CoNiCrFeCu-xSc HEAs.

Laser welding–brazing was performed using a YLS-5000 fiber laser (IPG, Oxford, MA, USA) with 5 kW power, a KR60 robot (KUKA, Augsburg, Germany), and a BIMOQ-BH (HIGHYAG, Kleinmachnow, Germany) laser head. Laser braze-welded joints were produced using a butt joint configuration with CoNiCrFeCu-xSc HEAs in between. A front and back shielding was supplied with a high-purity argon gas at a constant flow rate of 20 to 25 L/min to prevent the molten pool and heat-affected zone (HAZ) from oxidation. After mounting, grinding, and polishing, Murakami’s reagent was used to etch the laser braze-welded samples.

### 2.5. Bend Test and Fracture Analysis

Three-point bend test of joints was performed using a Zwick/Roell Z050 universal testing frame (Zwick, Ulm, Germany) equipped with an up to 50 kN load cell. The braze-welded specimens were cut into test coupons with dimensions of 80 × 4 × 3 mm. The length of the support span was 36 mm. All samples were tested in face-bend configuration at room temperature (20 °C) at a constant crosshead displacement velocity of 0.05 mm/min with a data acquisition rate of one sample per second. The flexural stress $\sigma_{f}$ and flexural strain $\varepsilon_{f}$ were calculated by:

(1) $$\sigma_{f}=\frac{3PL}{2bd^{2}}$$

(2) $$\varepsilon_{f}=\frac{6\delta d}{L^{2}}$$

where P is the measured load at fracture, L is the support span, b is the width, d is the section thickness, and δ is the measured deflection along the load direction at the mid-span. The fracture surfaces were characterized using a JSM–7800F field emission scanning electron microscope (JEOM, Tokyo, Japan).

## 3. Results

### 3.1. Microstructure and Phase Constituents of the CoNiCrFeCu-xSc HEAs

**Figure 1:**
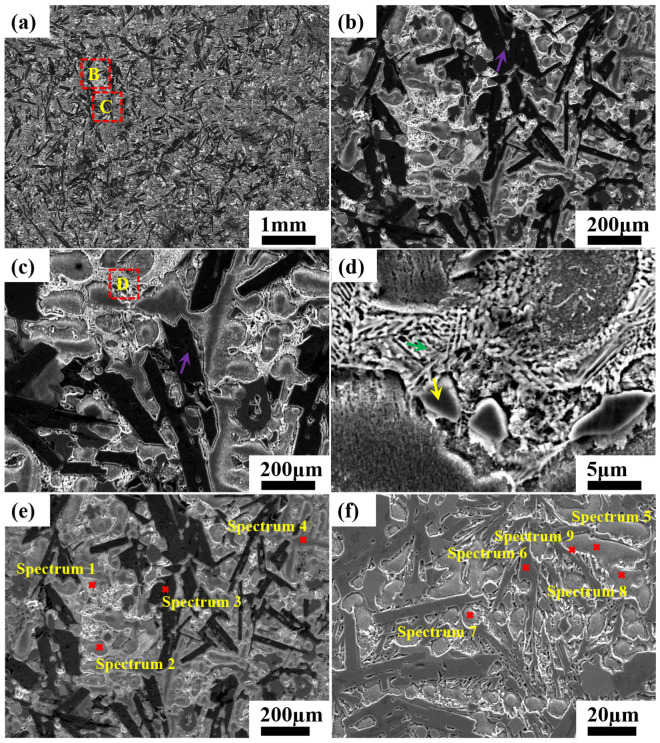
SEM micrographs of as-cast CoNiCrFeCu–0.5Sc HEAs, taken from a 10 mm-thick section. (**a**) Microstructure composed of a complex network of Ni–Si, elongated Cr–Fe–Co phase, and a gray Fe–Ni–Co–Cu solid-solution matrix. (**b**) Secondary electron image at higher magnification corresponding to the red rectangle “B” in (**a**), showing the typical elongated Cr–Fe–Co microstructure. (**c**) Higher-magnification image corresponding to the red rectangle “C” in (**a**), revealing the typical complex network Ni–Si phase (marked by purple arrows). (**d**) Higher-magnification image corresponding to the red rectangle “D” in (**c**), showing the complex network phase (green arrows) and gray Fe–Ni–Co–Cu solid-solution matrix (yellow arrows). Backscattered electron images showing EDS spectra locations: (**e**) Spectra 1–4 and (**f**) spectra 5–9 in the CoNiCrFeCu-0.5Sc HEA.

SEM images in Figure 1 show the typical microstructures of CoNiCrFeCu-xSc brazing filler metals. Backscattered electron images of labeled spectra 1 to 9 are shown in Figure 1e,f. SEM-EDS analyses of the labeled locations are shown in Table 3.

Figure 1 shows the typical microstructures of the HEAs. The microstructure is a mixture, consisting of three components: a complex network, an elongated Cr-Fe-Co, and a gray bulk matrix. A magnified view of region “B” in Figure 1a (see Figure 1b) shows the typical elongated Cr-Fe-Co phase in the matrix (as indicated by the purple arrow). The average width of the elongated phase is 83.3 µm. EDS analysis of the labeled location (spectra 3 and 6) reveals that the chemical composition of the elongated phase is 16.23% Co, 21.24% Fe, and 62.52% Cr (wt.%), which is similar to that of the Cr-Fe-Co phase reported in the literature.

The red rectangle region “C” in Figure 1a is magnified in Figure 1c, which shows the complex network is actually an eutectic structure. Further magnification in Figure 1d indicates that the complex network does indeed have a eutectic structure. According to the EDS analysis (spectrum 1) in Table 3, the eutectics consist of 15.75% Co, 35.86% Ni, 22.59% Si, 7.99% Sc, and the rest, including Fe, Cr, Mn, and Cu (wt.%). Therefore, the eutectics are suggested to comprise a component rich in nickel (cobalt) and silicon, and a component containing scandium (Ni–Si–Sc). In addition to reacting with nickel and cobalt to form compounds, scandium was also found to interact with copper, resulting in the formation of scandium copper phase, shown in spectrum 9, which reveals the chemical composition of a eutectic region to be 71.55% Cu, 9.46% Ni, 1.94% Sc, and the rest, including Co, Fe, Si (Sc-Cu).

The relatively high Si concentration observed in the network-like microstructure arises from both deliberate addition and severe elemental segregation during solidification, resulting in local co-enrichment of Si and Sc. Although the Cu content is higher than that of Sc in this localized region, Cu is homogeneously distributed within the FCC solid-solution matrix and shows no obvious segregation in the network structure. In contrast, Sc is significantly concentrated and combines with Si to form the key intermetallic compounds that dominate the network microstructure. For this reason, Sc rather than Cu is regarded as the main constituent element in this particular phase.

Figure 1d shows a magnified view of the red rectangle “D” in Figure 1c, the matrix of gray bulk Fe-Ni-Co solution (yellow arrow), at a higher magnification. The EDS spectra 2, 4, 5, 7, and 8 (Figure 1e,f) reveal the chemical composition of the matrix, which is 23.9% Co, 19.17% Ni, 23.82% Cu, 17.51% Fe, and the rest, including Cr, Mn, and Si. The matrix is suggested to be a solid solution that mainly includes Co, Cu, Ni, and Fe.

**Figure 2:**
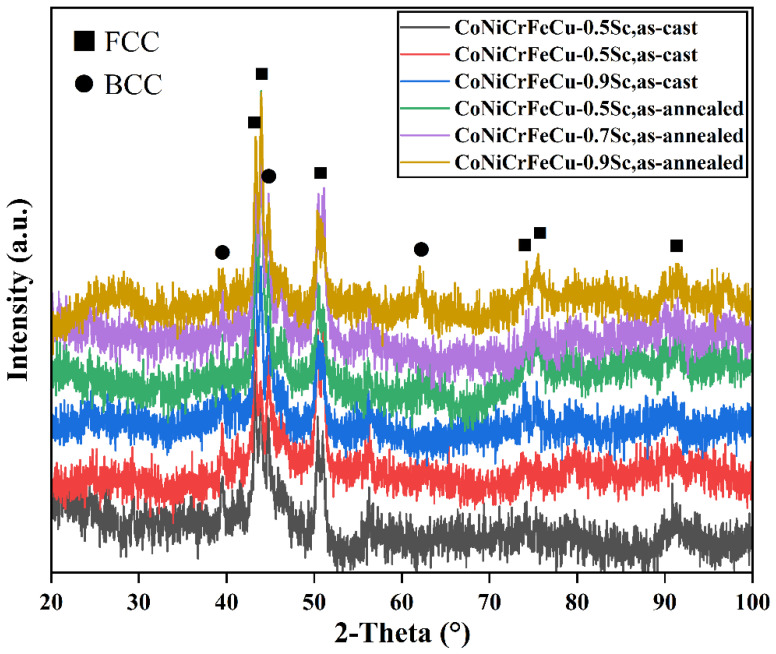
XRD patterns of CoNiCrFeCu-xSc (x = 0.5, 0.7, 0.9) high-entropy alloys.

The phases present in the CoNiCrFeCu-xSc (x = 0.5, 0.7, 0.9 wt.%) HEAs were identified by XRD analysis using longitudinal specimens sectioned parallel to the diameter of the disk-shaped ingots. Specimens in both the as-cast and annealed states were examined to obtain representative X-ray diffraction patterns (Figure 2). In the CoNiCrFeCu-xSc HEAs, a dominant FCC phase and a minor BCC phase were detected. The FCC phase was rich in Co-Cu, Cu-Ni, Cu-rich, Cr-Fe-Co, and Ni-Sc, while the BCC phase was Cr-rich, if the peak positions of the main FCC phase were carefully observed. Some of the eutectic phases were not observed, likely due to their small amount. However, these smaller amounts of phases are characterized next at higher magnification under TEM.

### 3.2. Structural Observations by TEM and Elongated Cr-Fe-Co

**Figure 3:**
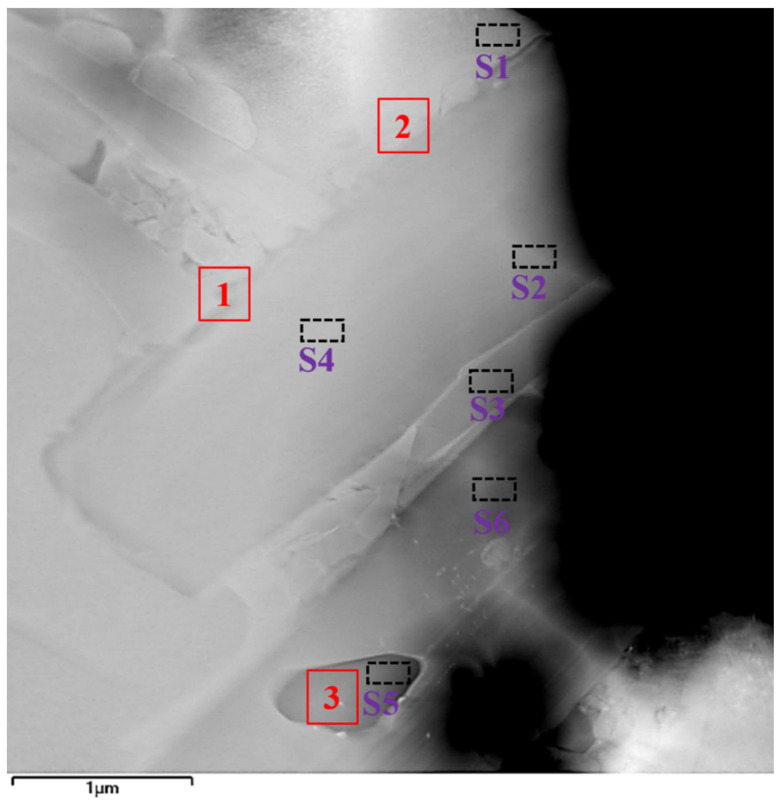
TEM-EDS analysis of CoNiCrFeCu-0.9Sc HEAs showing the typical elongated Cr-Fe-Co phase, a Cr-rich phase, and a cobalt matrix. Results of EDS analysis (spectra S1–S6) of the labeled locations are shown in Table 4. Bright-field images, SAED images, and high-resolution crystal lattice of regions “1”, “2”, and “3” are shown in Figure 4.

Figure 3 shows the location of TEM and EDS analysis obtained from the CoNiCrFeCu-0.5Sc HEAs. Three typical microstructures, Cr-Fe-Co, Co matrix, and Cr-rich phase, were selected for further TEM analysis, regions “1”, “2”, and “3”, as indicated by the red rectangle. For further investigation, spectra S1–S6 were selected for further EDS analysis. Results of EDS analysis (spectra S1–S6) of the labeled locations are shown in Table 4. The results indicated that cobalt, iron, and nickel dominated spectrum S1. However, the energy spectra of points S2, S3, S4, and S6 indicated that they were primarily composed of Cr-Fe-Co solid solution. Scandium was observed in point S3. The energy spectra of point S5 indicated that the point was a Cr-rich phase.

**Figure 4:**
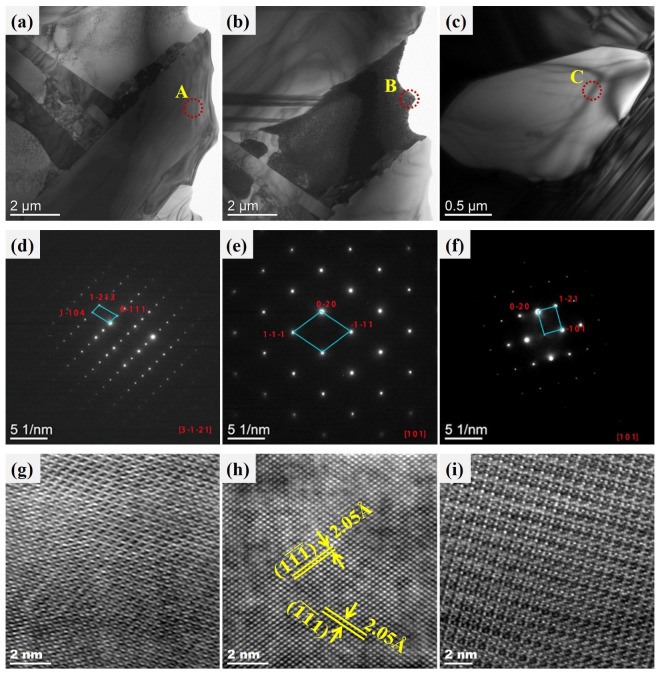
STEM observations of typical elongated microstructure and crystal structure analysis by high-resolution TEM and indexed by selected area electron diffraction (SAED) pattern (inset). Bright-field images of (**a**) region “1”, (**b**) region “2”, and (**c**) region “3” in Figure 3. The selected area electron diffraction (SAED) patterns for regions “A”, “B”, and “C” are presented in (**d**), (**e**), and (**f**), respectively. High-resolution crystal lattice showing (**g**) in the region is indicated by a yellow round dot lines area “A”. (**h**) Co-FCC in the region indicated by a yellow round dot lines area “B”. (**i**) The lattice crystal and Cr-BCC in the region indicated by a yellow round dot lines area “C”.

Figure 4 shows a magnified bright-field TEM image of the selected region, revealing three types of features: a typical elongated Cr-Fe-Co phase, a dominant Co matrix, and the Cr-rich phase, which are indicated by the red rectangles “1”, “2”, and “3”, respectively. Scanning transmission electron microscopy (STEM) images of typical phases are shown in Figure 4a–c. Three regions indicated by yellow dot circles “A”, “B”, and “C” were selected for the selected area electron diffraction (SAED) patterns and high-resolution TEM.

As shown in Figure 4a, the elongated Cr-Fe-Co structure is a mixture where Cr is present in the Fe-Co solid solution. This feature is confirmed by the SAED pattern, as well as the high-resolution TEM image shown in Figure 4d. The structure of the dominant Co matrix is determined by SAED patterns (inset in Figure 4b). Along the crystallographic zone axis direction of [111], the crystal planes $\left(1\bar{1}\bar{1}\right)$, $(\bar{1}\bar{1}1$) and $\left(0\bar{2}0\right)$ were observed. The d-spacing of 2.05 Å was calculated through characterization using high-resolution TEM in Figure 4e. It represents a Cr-Fe-Co solid solution with an FCC structure. The Cr-rich phase is confirmed to be BCC chromium by the SAED inset in Figure 4c and high-resolution TEM in Figure 4f.

**Figure 5:**
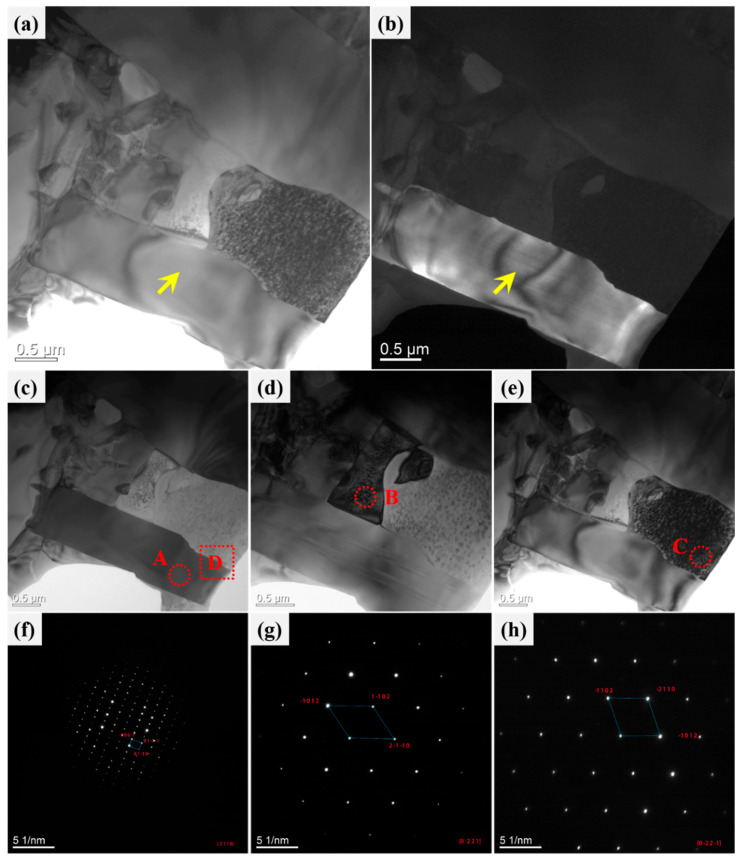
TEM images of CoNiCrFeCu-0.9Sc HEAs. (**a**) Bright-field and (**b**) dark-field TEM images showing Ni_2_Si precipitation (yellow arrow). (**c**) Region “A” denotes Ni_2_Si precipitation. (**d**) Region “B” represents FCC-Cu. (**e**) Region “C” is FCC-Ni, SAED patterns of (**f**) region “A” in (c). (**g**) region “B” in (d). (**h**) region “C” in (**e**).

The bright-field TEM image of nickel, silicon, copper, and their chemical compounds is shown in Figure 5a. The bright-field image reveals three typical crystal structures in the selected area, labeled as positions 1, 2, and 3 with yellow rectangles in the image. The corresponding dark-field image is shown in Figure 5b.

In the TEM-SAED analysis presented in Figure 5, Figure 5c–e shows the bright-field images of regions “A,” “B,” and “C,” respectively. The corresponding SAED patterns for these three regions are shown in Figure 5f–h. Among them, the region “A” shown in Figure 5c is also the precipitate observed in Figure 5b. With [$\bar{2}110]$ as the zone axis, the diffraction pattern for region “A” is shown in Figure 5f. By comparing the acquired electron diffraction pattern with the standard reciprocal lattice (standard diffraction pattern), the indices of each diffraction plane can be directly determined: $\left(000\bar{1}\right)$, $\left(01\bar{1}\bar{1}\right)$, and $\left(01\bar{1}0\right)$. Thus, it can be concluded that the crystal structure of region “A” is hexagonal-Ni_2_Si (cell: 3.797×3.797×4.8928). Ni_2_Si is a metastable phase with a non-equilibrium crystal structure. It belongs to the category of intermetallic compounds and is composed of nickel (Ni) and silicon (Si) elements. Figure 5d shows the region “B” with [$0\bar{2}21]$ as the zone axis, and the corresponding diffraction pattern for region “B” is shown in Figure 5g. This diffraction pattern confirms that the crystal structure of region “B” is FCC-Cu. Figure 5e displays the region “C” with [$0\bar{2}2\bar{1}]$ as the zone axis, and the diffraction pattern for region “C” is shown in Figure 5h. This diffraction pattern confirms that the structure of region “C” is FCC-Ni.

### 3.3. Laser Welding–Brazing of WC-Co and Steels Using CoNiCrFeCu-0.5Sc Filler

**Figure 6:**
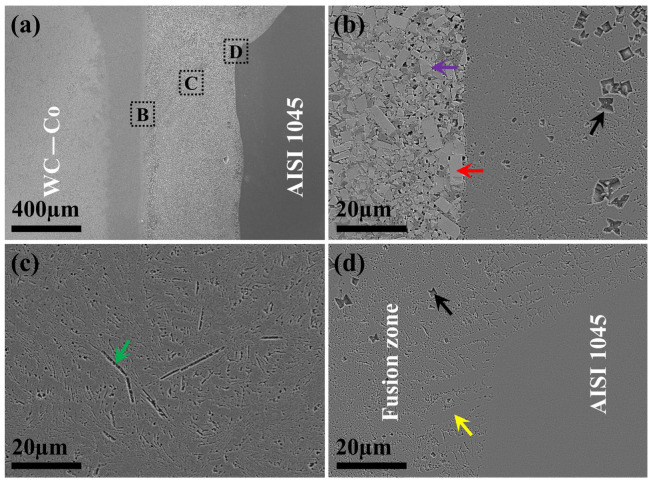
Microstructure of the braze-welded joints of WC-20Co and AISI 1045 steel. (**a**) Cross-sectional view of a typical joint (V8) of WC-20Co (left side) and AISI 1045 (right side). (**b**) WC–Co/fusion zone interface, region “B” in (**a**). (**c**) Fusion zone, region “C” in (**a**). (**d**) Fusion zone/AISI 1045 interface, region “D” in (**a**).

The SEM image in Figure 6a shows the microstructure of the braze-welded joints: WC–Co/weld interface, fusion zone, and weld/AISI 1045 interface. The typical joint in cross-sectional view consists of a straight fusion boundary on the WC-Co side and a nail-head-shaped weld fusion boundary on the steel side. There is a greater dilution from the top side than from the lower side of the steel substrate.

An SEM image of the WC-Co side fusion boundary region (indicated by HAZ in Figure 6a) is shown in Figure 6b. The tungsten carbide (WC) grains on the left side of the figure appear to have retained their cubic shape (indicated by the red arrow) or triangular shape (indicated by the purple arrow) in the binder matrix. The fusion zone itself appears to have solidified in eutectic microstructure with the embedded particles (indicated by the black arrow). As shown in Figure 6c, in the fusion zone, the eutectic microstructure has a coarse needle-like phase (indicated by the green arrow). The fusion boundary on the steel side (Figure 6d) appears to have large primary dendrites epitaxially grown from the steel substrate (indicated by the yellow arrow).

### 3.4. Bend Strength and Fractography

Due to the statistical nature of the mechanical properties of the brazed joints, three repeat specimens were bend-tested for each brazed sample. Table 5 summarizes the bend strength for three-point bend tests of the samples. For the 3 mm-thick samples 1-1, 1-2, and 1-3, they were brazed with CoNiCrFeCu-0.5Sc HEA as the brazing filler metal. Three specimens exhibited linear stresses with bend strengths between 182.3 and 243.2 MPa.

With the same laser input (Sc elemental content in the brazing filler metals was increased from 0.5 wt.% to 0.7 wt.%), 3 mm-thick specimens showed higher bend strength between 307.2 and 381.2 MPa (Table 5, samples 2-1, 2-2, and 2-3). Samples 3-3 in Table 5 showed that braze-welded joints with 0.9 wt.% Sc had significant bend strength and ductility, with a maximum strength of 411.5 MPa. It is confirmed that the increased Sc level improves the flexural strength of the braze-welded joints.

**Figure 7:**
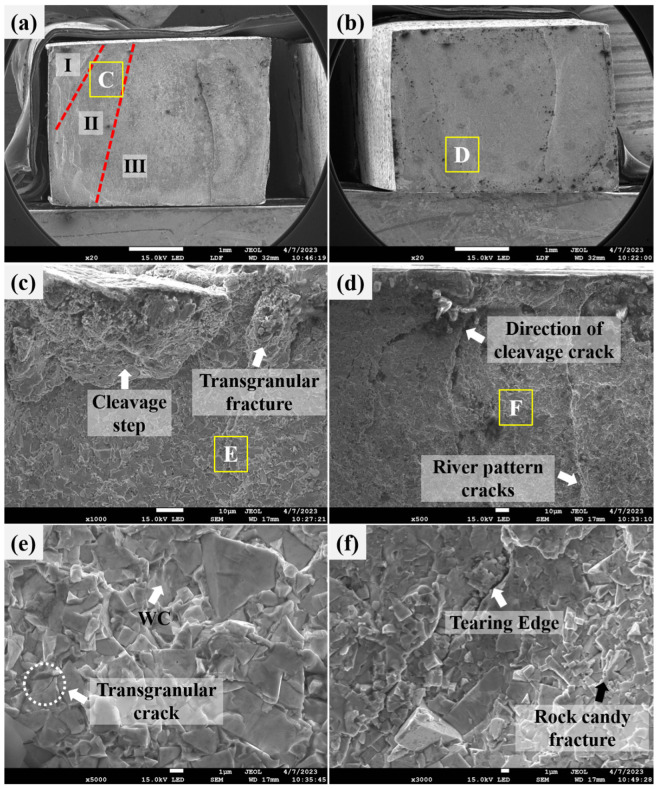
Specimen showing the fracture path of the cleavage surface for the typical face-bend test. (**a**) WC-Co side. (**b**) Braze-welded side. Typical bend fracture surfaces (**c**). Transverse granular fracture within the fusion zone (white rectangular area “C” in (**a**)), forming a cleavage step; (**d**) white area “D” (see (**b**)) cleavage fracture direction, forming a “river” fracture; (**e**) yellow rectangular area “E” (see (**c**)) showing undissolved tungsten carbide particles, and trans-granular crack (**f**) enlargement of yellow rectangular area “F” showing the fracture surface in the tearing edge rock candy fracture.

All bending test specimens fractured along the heat-affected zone (fusion boundary) on the WC-Co side. The fracture surface (Figure 7a) exhibits different fracture modes, characterized by a combination of brittle and ductile fracture, which helps determine the fracture characteristics and failure mechanisms of the braze-welded joint. The three main regions of a fracture surface are: Zone I → Zone II → Zone III (WC–Co side). In zone I, the surface is rough during loading, and many slight cracks appear.

As the load continues to increase, the cracks begin to develop into a major source of cracks and extend to zone II. After reaching a certain load level, the cracks rapidly extend to zone III, leading to a brittle failure of the braze-welded joint. Figure 7b shows the fusion zone near the brazed filler metal side.

Figure 7c shows a typical fracture surface (white rectangular area “C” in Figure 7a). In the weld fusion zone of the fracture, the fracture mode is an along-crystal fracture with the formation of disintegration steps (Figure 7c). In the HAZ part of the fracture, the fracture mode is an intergranular fracture. At higher magnification, the joint fracture morphology shows a river pattern. Figure 7d shows a further magnification of the white rectangular area “D” in Figure 7b, which is the direction of convergence and crack extension of the direction of cleavage crack, and the upstream direction of the river is the direction of crack source generation. The fracture in Figure 7e (yellow rectangular area “E” in Figure 7c) shows that the undissolved WC particles are separated by cleavage, and this area also shows small cracks in solution, which show some local ductility, as shown in Figure 7e. Figure 7f shows a further enlargement of the yellow rectangular region “F” (Figure 7d), which produces tearing edges with rock candy fracture.

## 4. Discussion

### 4.1. Effect of Scandium on the Melting Characteristics of CoNiCrFeCu-xSc

**Figure 8:**
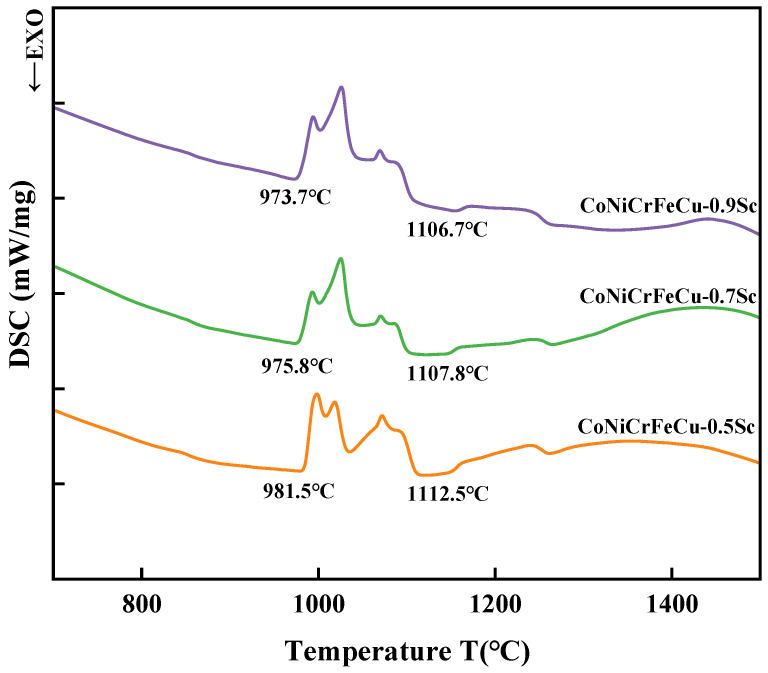
TG-DSC curve of CoNiCrFeCu-xSc HEAs at a heating rate of 20 °C/min.

Figure 8 shows the TG-DSC curves of the CoNiCrFeCu-xSc HEAs at a heating rate of 20 °C/min. The statistical results of the solidus, liquidus, and the melting range of the three HEAs are shown in Table 6. For brazing filler metal with a scandium content of 0.5 wt.% (CoNiCrFeCu-0.5Sc), the brazing filler metal starts melting when the temperature increases to 981.5 °C, and completely liquefies as soon as the liquidus temperature (1112.5 °C) has been reached. With the increase in scandium, the solidus and liquidus temperatures of the HEAs decrease, and the temperature range between the solidus and liquidus increases.

During the heating process, the HEAs undergo a grain growth process. Several peaks are observed in the solid and liquid temperature ranges, indicating that the alloys undergo phase transformation during melting.

### 4.2. Effect of Sc on the Wettability of CoNiCrFeCu-xSc

**Figure 9:**
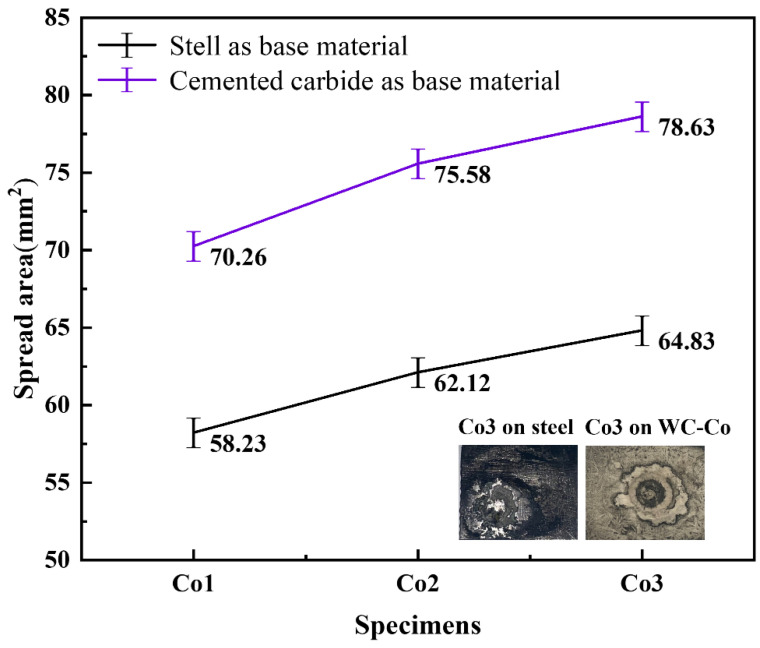
Spreading area of CoNiCrFeCu-xSc on S1045 carbon steel and WC-Co hard metals.

The wettability of the HEAs to the base metals is crucial to the formation and mechanical properties of the braze-welded joint. The spreading experiment was carried out in a high-frequency induction furnace with an output current of 403A and lasted for 30 s to ensure complete melting of the HEAs. Figure 9 shows spreading areas of CoNiCrFeCu-xSc HEAs on the base materials: AISI 1045 carbon steel and WC-Co hard metal. The results indicated that the CoNiCrFeCu-xSc HEAs with different Sc contents showed excellent spreading properties on both WC-Co hard metal and carbon steel. The spreading properties of the CoNiCrFeCu-xSc on WC-Co hard metal were better than those on steel. CoNiCrFeCu-0.5Sc brazing filler metal has a spreading area of 58.23 mm^2^ on AISI 1045 carbon steel and 70.26 mm^2^ on WC-Co hard metal.

With the increase of scandium content in CoNiCrFeCu-xSc HEAs, the spreading area of the CoNiCrFeCu-xSc HEAs on both materials increases. When the scandium content in CoNiCrFeCu-xSc was increased from 0.5 wt.% to 0.7 and 0.9 wt.%, the spreading areas were 62.12 mm^2^, 64.83 mm^2^ on AISI 1045 steel and 77.58 mm^2^, 78.63 mm^2^ on WC-Co hard metal. The maximum spreading area on the AISI 1045 steel surface is 64.83 mm^2^, reaching 78.63 mm^2^ on the WC-Co hard metal.

### 4.3. Effect of Sc on the Micro Hardness of CoNiCrFeCu-xSc

Hardness testing was employed to measure the hardness of CoNiCrFeCu-xSc high-entropy alloys (HEAs). Twelve hardness values were randomly measured at different locations on the sample, and the highest and lowest values were discarded. The average of the remaining ten data points was taken as the micro hardness value of the CoNiCrFeCu-xSc HEAs.

The micro hardness value of CoNiCrFeCu-xSc HEAs with a scandium content of 0.5% is 263.5 HV. As the scandium increases from 0.5% to 0.7%, the micro hardness of CoNiCrFeCu-xSc HEAs significantly increases, reaching 300.2 HV. However, as the scandium further increases from 0.7% to 0.9%, the micro hardness decreases to 291.7 HV. Therefore, within the range of scandium in this study, the micro hardness increases initially and then tends to slightly decrease with the increase in scandium. The maximum value of 300.2 HV is achieved when the scandium is 0.7%.

The effect of scandium on the micro hardness of CoNiCrFeCu-xSc HEAs can be summarized as follows: (1) Alloy strengthening: Scandium reacts with nickel to form Ni-Sc compounds, and with nickel and silicon to form Ni_16_Sc_6_Si_7_ compounds. These compounds form fine precipitates within the nickel matrix, contributing to increased micro hardness; and (2) hindering dislocation movement: the compounds and solid solution formed by scandium increase the interfaces in the CoNiCrFeCu-xSc HEAs. Multiple interfaces impede the movement and slip of dislocations [44,45], thus increasing the alloy’s hardness. The interfaces act as barriers to dislocation motion, restricting their movement within the CoNiCrFeCu-xSc HEAs.

However, when the scandium in CoNiCrFeCu-xSc HEAs increases to 0.9 wt.%, the hardness no longer increases and instead shows a slight decrease due to the formation of brittle phases, large precipitates, and distortion of the crystal lattice. Therefore, the optimal Sc addition for achieving the highest micro hardness in CoNiCrFeCu-xSc HEAs is 0.7 wt.%.

### 4.4. The Behavior of Chromium and Scandium in CoNiCrFeCu-xSc HEAs

**Figure 10:**
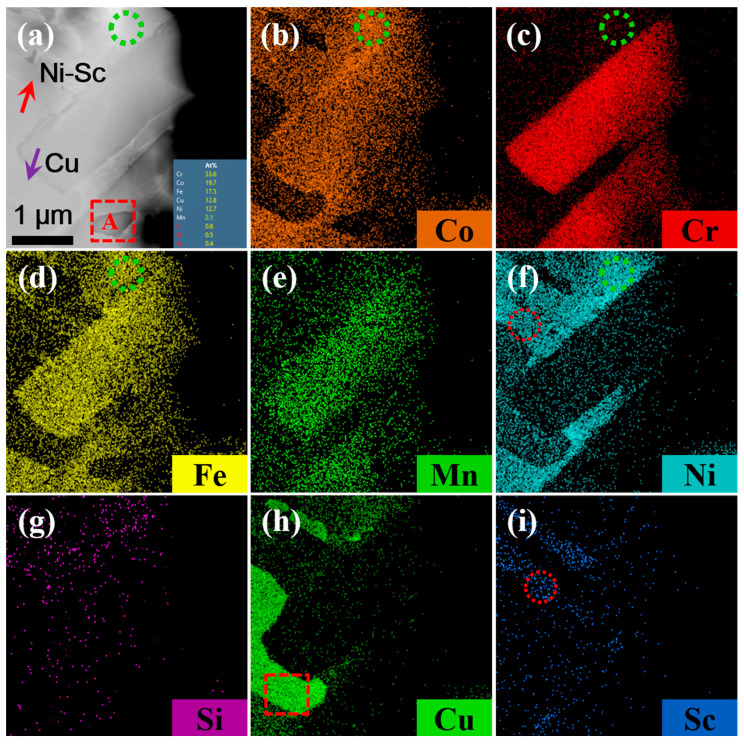
Scanning transmission electron microscopy (STEM) observations and energy-dispersive X-ray spectroscopy (EDS) mapping. (**a**) A Cr-Co-Fe-Mn-rich mixed phase surrounded by Ni-Sc (red arrow) and Cu (purple arrow), and EDS map of elements (**b**) Co, (**c**) Cr, (**d**) Fe, (**e**) Mn, (**f**) Ni, (**g**) Si. (**h**) Cu, and (**i**) Sc.

Chromium exists in three forms in CoNiCrFeCu-xSc HEAs. The first form is a black elongated microstructure of Cr-Fe-Co mixture (Chromium: 62.52%), as shown in Figure 1b, Figure 1 (spectra 3 and 6), and Table 3. TEM image (region “1” in Figure 3), BF TEM image (Figure 4a and the inset), SAED (Figure 4d), and the corresponding TEM-EDS analysis results (Table 4) confirm that chromium exist as Cr-BCC and the black elongated microstructure is mainly a mixed phase formed by Cr elements with the rest of the amounts of Fe and Co. From STEM-EDS mapping (Cr) in Figure 10, copper, silicon, nickel, scandium is not observed in the black elongated microstructure. A small black elongated bridge appears between the adjacent black elongated microstructures. They consist of Cr, Fe, and Co. In addition to these three elements, STEM observations and EDS mapping also confirmed the existence of manganese in very small amounts.

**Figure 11:**
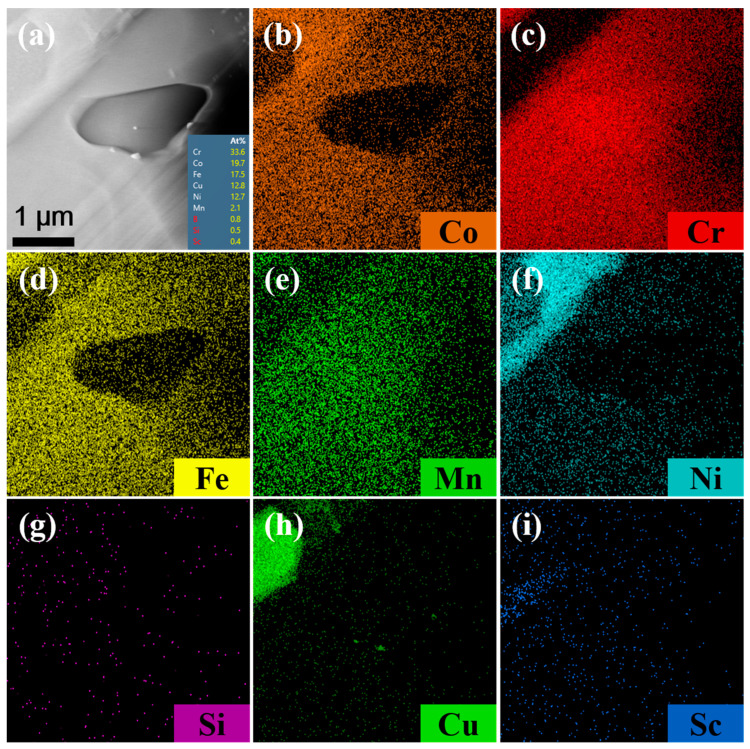
STEM observations and EDS mapping of the Cr-rich microstructure. (**a**) A Cr-rich phase (region “3”, Figure 3 and Figure 6c), indicated by the red dot rectangle “A” in Figure 10a and EDS map of elements (**b**) Co, (**c**) Cr, (**d**) Fe, (**e**) Mn, (**f**) Ni, (**g**) Si. (**h**) Cu, and (**i**) Sc.

The second form is an elliptical-shaped chromium-rich phase with a chromium content of 91.60%, as shown in Figure 3 (region “3” in Figure 3), BF TEM image (Figure 4c and the inset), SAED (Figure 4f), and Table 4. Figure 11 shows STEM images and EDS mapping of the Cr-rich microstructure shown in Figure 10a. This means part of the Cr-rich should be due to segregation.

The third form of chromium is present as a gray bulk with chromium dissolving into the Fe-Ni-Co matrix. In this form, chromium is present in very small amounts, as shown in Figure 1e,f (Spectra 2, 4, 5, 7, and 8) and Table 3. TEM image (Spectra S1 in Figure 3) and TEM-EDS analysis results (Table 4) confirm the results. According to the STEM image and EDS mapping of the gray bulk indicated by green dot circles, the gray bulk contains Fe, Ni, Co, and small amounts of chromium.

Therefore, in CoNiCrFeCu-xSc HEAs, forms of chromium contain: (1) Alloy phase: chromium exists in solid solution within alloys, forming a solid-solution Cr-Fe-Co phase with iron and cobalt. In this form, chromium accounts for over 60% of the phase. (2) Chromium remains undissolved and exists in its elemental form. This form is common in strengthening, the formation of stable phases, and other applications. In this form, chromium accounts for more than 90% of the phase. (3) Dissolved state: Chromium can exist in a dissolved state in the Fe-Ni-Co matrix. In this form, chromium accounts for less than 10% of the phase.

Scandium was found at the interface between the Cr-Fe-Co mixture and Fe-Ni-Co matrix, as shown in Figure 2 (spectra 1 and 9) and Table 3. Scandium is a soft, silvery transition metal, and an appropriate amount of scandium can improve the spreading areas of CoNiCrFeCu-xSc HEAs (Figure 9). The addition of scandium can also modulate the alloy’s lattice structure and grain size [46], thereby influencing the physical and mechanical properties of the alloy [47,48,49]. The region indicated by the red arrow contains Ni-Sc alloy, and the mapping of scandium and nickel in Figure 10f,i (indicated by red dotted circles), confirms their existence. Scandium can be dispersed within the matrix nickel, enhancing the mechanical properties of the alloy through interactions with the matrix nickel [43]. According to the research proposed by Maslenkov et al. [50], three chemical compounds: ScNi_5_, Sc_2_Ni_7_, and ScNi_2_ can be formed in the Ni-Sc system. The ratio of Sc and Ni is calculated, in this study, to be 16:6, as shown in Figure 2. So, Sc may be part of these three compounds. Moreover, scandium can dissolve in the nickel (spectrum 9 in Figure 1e,f and Table 3) or Cr-Fe-Co matrix (Figure 3 and Table 4) as a solid solution, improving the strength and hardness through solid-solution strengthening mechanisms.

### 4.5. Interface

**Figure 12:**
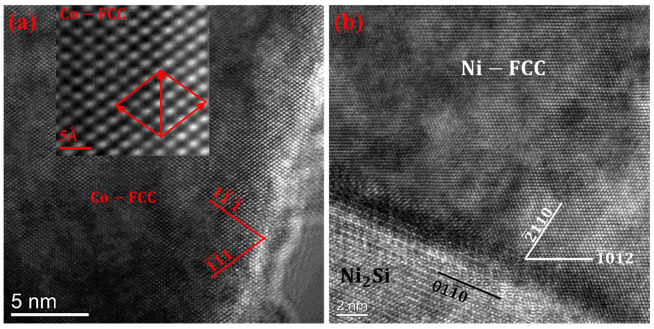
HRTEM image of (**a**) Co/Co interface and (**b**) $\mathrm{Ni}/{\mathrm{Ni}}_{2}\mathrm{Si}$ interface showing slip deformation, stacking faults, and dislocations.

Figure 12a shows a high-resolution TEM image of region “B” in Figure 4b, and a further magnified view of region D is shown in the inset of the Figure. It can be observed that the typical crystal planes include $\left(1\bar{1}\bar{1}\right)$ and $\left(\bar{1}\bar{1}1\right)$, and combining with Figure 4, this interface represents the Co/Co interface. Figure 12b displays a high-resolution TEM image of region “D” in Figure 5c, which represents a typical Ni_2_Si/Ni interface.

As shown in Figure 3 and Figure 12b, there are numerous grain boundaries, dislocations, and microstructural defects present in the crystal structure of CoNiCrFeCu-xSc HEAs. These defects can impede the motion of dislocations and crystal slip, thereby enhancing the strength and toughness of the alloy [51,52].

In the TEM images (Figure 4, Figure 5 and Figure 12), crystal structures of FCC-Co, BCC-Cr, FCC-Cu, and FCC-Ni were identified, demonstrating the presence of multiple grain boundaries, phase boundaries, or interfaces in CoNiCrFeCu-xSc HEAs. The existence of multiple interfaces can create boundaries between different phases, enhancing the strength and hardness of CoNiCrFeCu-xSc HEAs through the strengthening effect of phase boundaries. These phase boundaries can impede the movement of dislocations, increasing the energy consumption of dislocations and thereby improving the deformation resistance of CoNiCrFeCu-xSc HEAs. The presence of multiple interfaces increases the number of grain boundaries, limiting the movement of dislocations in the crystal [53,54] and making plastic deformation more difficult. Multiple interfaces play various key roles in CoNiCrFeCu-xSc HEAs, influencing the alloy’s mechanical properties, structural stability, and thermodynamic behavior [55,56,57].

Commercially, H62 brass and HL105 filler are among the most successful brazing materials currently used for joining cemented carbide and steel [58]. For cemented carbide–steel components used in aircraft, vacuum brazing is generally required [59]. In cutting pick machines, cemented carbide and steel are reliably joined by high-frequency induction brazing with Cu-based alloys, combined with preheating and post-weld heat treatment [9]. As a clean, simple, and rapid approach, induction brazing is widely used for mass production. For cemented carbide components intended for high-temperature service, such as valve parts, Ni-based fillers are frequently employed together with sinter-bonding technology to join cemented carbide and steel [60].

A quantitative comparison reveals that compared with Ni-based fillers [9], the proposed CoNiCrFeCu-xSc alloy enables a lower brazing temperature, and its multi-component alloying design avoids the brittle risk of coarse M_6_C and M_12_C complex carbides. Although the Ag-Cu-Ti filler developed by Zheng et al. [13] showed a lower brazing temperature, the joints brazed at 900 °C for 10 min fractured in the middle of the brazing seam with an average shear strength of 235 MPa. In contrast, the CoNiCrFeCu-xSc filler in this work, brazed at approximately 1000 °C, achieved significantly higher bonding strength, hardness, and service temperature stability; moreover, the introduction of laser improves processing efficiency. Compared with previously reported HEAs [7,12,27,29], our designed CoNiCrFeCu-xSc possesses a lower brazing temperature, and the addition of Sc enhances its braze-ability and wettability. Overall, the developed HEAs exhibit a more balanced combination of strength and ductility relative to conventional fillers.

## 5. Conclusions

CoNiCrFeCu-xSc (x = 0.5, 0.7, 0.9 wt.%) HEAs were prepared using a high-vacuum non-consumable arc melting process. After materials characterization and measuring the wettability and melting point, the possibility of using CoNiCrFeCu-xSc high-entropy alloys (HEAs) as the filler for laser-welding brazing of WC-Co hard metals to steels was explored. The following conclusions can be drawn:

1. The HEAs exhibit a complex microstructure characterized by the coexistence of elemental phases, intermetallic compounds, and eutectics, with a uniform distribution of constituent elements. This microstructural integrity provides excellent thermal stability. TG-DSC analysis determined the melting range of the filler materials to be between 973.7 °C and 981.5 °C.
2. The HEAs demonstrate high wettability on both WC-Co cemented carbide and AISI 1045 steel surfaces. The spreading area increases proportionally with scandium (Sc) content. At a Sc concentration of 0.9 wt.%, the maximum spreading areas reached 64.83 mm^2^ on AISI 1045 steel and 78.63 mm^2^ on WC-Co.
3. SEM, XRD, and TEM analyses indicate that the system consists of a dual-phase structure comprising FCC and BCC phases. The FCC phase is predominantly enriched in Co-Cu, Cu-Ni, Cr-Fe-Co, and Ni-Sc, while the BCC phase is Cr-rich.
4. Scandium exists within the HEAs primarily as a solid solution and in the form of chemical compounds. The addition of Sc narrows the melting temperature range, lowers the solidus temperature of the filler metals, and enhances the wetting behavior critical for sound braze-welded bond formation.

## Figures and Tables

**Table 2:** Laser brazing parameters using CoNiCrFeCu-xSc HEAs as brazing filler metals.

Specimens	Laser Power (P, kW)	Scanning Rate (v, m/s)	Defocus Amount (f, mm)	Thickness (d, mm)	Filler
V_1_	1.23	0.016	−4	0.5	Co1
V_2_	1.23	0.016	−8	0.5	Co1
V_3_	1.4	0.016	−8	0.5	Co1
V_4_	1.4	0.014	−8	0.5	Co1
V_5_	1.4	0.010	−8	0.5	Co1
V_6_	1.4	0.012	−8	0.5	Co1
V_7_	1.4	0.012	−8	0.5	Co2
V_8_	1.4	0.012	−8	0.5	Co3

**Table 3:** EDS-measured chemical compositions (at.% and wt.%) of the marked regions in Figure 1e,f.

Point	Cobalt	Nickel	Copper	Iron	Chromium	Silicon	Manganese	Scandium
Spectrum 1	(18.30) (15.75)	(41.50) (35.86)	(10.13) (08.09)	(04.89) (04.44)	(02.39) (02.34)	(12.51) (22.59)	(03.20) (02.95)	(07.08) (07.99)
Spectrum 2	(25.13) (23.90)	(20.08) (19.17)	(27.01) (23.82)	(17.51) (17.51)	(04.13) (04.45)	(04.94) (09.86)	(01.20) (01.22)	—
Spectrum 3	(17.73) (16.23)	—	—	(22.00) (21.24)	(60.27) (62.52)	—	—	—
Spectrum 4	(27.14) (25.79)	(20.00) (19.08)	(24.64) (21.71)	(18.27) (18.32)	(05.17) (05.57)	(04.78) (09.52)	—	—
Spectrum 5	(24.78) (23.53)	(17.82) (16.98)	(27.12) (23.88)	(20.90) (20.594)	(04.39) (04.72)	(05.00) (09.96)	—	—
Spectrum 6	(22.47) (20.76)	—	—	(26.82) (26.15)	(50.71) (53.10)	—	—	—
Spectrum 7	(25.67) (24.47)	(20.87) (19.97)	(24.04) (21.25)	(19.79) (19.91)	(05.28) (05.70)	(04.35) (08.70)	—	—
Spectrum 8	(23.28) (22.42)	(20.52) (19.84)	(27.79) (24.83)	(19.66) (19.99)	(05.13) (05.60)	(03.62) (07.33)	—	—
Spectrum 9	(05.62) (05.67)	(09.35) (09.46)	(76.54) (71.55)	(03.28) (03.49)	—	(03.73) (07.88)	—	(01.47) (01.94)

**Table 4:** TEM-EDS-determined composition of locations labeled in Figure 3 (wt.%).

Point	Co	Cr	Fe	Ni	Cu	Si	Mn	Sc
S1	(31.30)	(4.50)	(23.20)	(26.90)	(9.60)	(1.60)	(2.30)	—
S2	(16.20)	(58.80)	(20.10)	(2.10)	(0.40)	(0.20)	(1.50)	—
S3	(22.00)	(47.00)	(23.20)	(3.60)	(0.40)	(0.20)	(1.80)	(0.10)
S4	(16.60)	(58.80)	(20.10)	(2.00)	(0.40)	(0.20)	(1.50)	—
S5	(2.30)	(91.60)	(4.00)	(0.60)	(0.30)	(0.20)	(0.90)	—
S6	(16.20)	(58.70)	(20.3)	(1.80)	(0.30)	(0.20)	(1.60)	—

**Table 5:** Flexural strength of the braze-welded samples.

Co1	$\sigma_{b}$ (MPa)	Co2	$\sigma_{b}$ (MPa)	Co3	$\sigma_{b}$ (MPa)
1-1	243.2	2-1	362.2	3-1	399.6
1-2	231.6	2-2	381.2	3-2	408.6
1-3	182.3	2-3	307.2	3-3	411.5

**Table 6:** Melting temperature and melting range of CoNiCrFeCu-xSc HEAs.

HEAs	Solidus (°C)	Liquidus (°C)	Melting Range (°C)
CoNiCrFeCu-0.5Sc	981.5	1112.5	131
CoNiCrFeCu-0.7Sc	975.8	1107.8	132
CoNiCrFeCu-0.9Sc	973.7	1106.7	133

## Data Availability

The original contributions presented in this study are included in the article. Further inquiries can be directed to the corresponding authors.
